# Clonal Hematopoiesis From Next Generation Sequencing of Plasma From a Patient With Lung Adenocarcinoma: A Case Report

**DOI:** 10.3389/fonc.2020.00113

**Published:** 2020-02-13

**Authors:** Munehiro Ito, Yutaka Fujiwara, Takashi Kubo, Hiromichi Matsushita, Tadashi Kumamoto, Tatsuya Suzuki, Kuniko Sunami, Noboru Yamamoto, Takashi Kohno

**Affiliations:** ^1^Department of Thoracic Oncology, National Cancer Center Hospital, Tokyo, Japan; ^2^Department of Experimental Therapeutics, National Cancer Center Hospital, Tokyo, Japan; ^3^Department of Respiratory Medicine, Mitsui Memorial Hospital, Tokyo, Japan; ^4^Division of Translational Genomics, Exploratory Oncology Research and Clinical Trial Center, National Cancer Center, Tokyo, Japan; ^5^Department of Laboratory Medicine, National Cancer Center Hospital, Tokyo, Japan; ^6^Department of Pediatric Oncology, National Cancer Center Hospital, Tokyo, Japan; ^7^Department of Hematology, National Cancer Center Hospital, Tokyo, Japan; ^8^Division of Genome Biology, National Cancer Center Research Institute, Tokyo, Japan

**Keywords:** cell-free DNA, clonal hematopoiesis, next generation sequencing, precision medicine, TP53 mutation

## Abstract

Reliable and accurate next generation sequencing (NGS) technologies are important in precision medicine. Analysis using currently available NGS genomic tests is conducted on cancer-derived DNA collected from tumor tissue, blood, or both. Clonal hematopoiesis (CH) produces a detectable somatic clonal mutation that is commonly associated with clonal expansion of hematopoietic cells with age and genomic analysis of blood samples can be used to detect CH. A 74-year-old Korean male had lung adenocarcinoma with a metastasis to the left scapula. He underwent palliative radiotherapy to the left scapula and received multi-line chemotherapies. After disease progression, he underwent re-biopsy of the metastatic tumor tissue from lung cancer and concomitant blood sampling. NGS genomic testing revealed no significant genomic mutation in the tumor tissue DNA but showed the *TP53* mutation C135Y in peripheral blood DNA. To investigate the discordance between the genotyping results in tumor tissue and blood, we tested for the *TP53* mutation using a target sequencing test in blood and normal oral mucosa. The *TP53* mutation C135Y was only detected in the blood sample, confirming the presence of *TP53*-mutated CH. We should be aware of different characteristics in NGS genomic testing including sample type such as tumor, blood, or paired specimens. Performing genomic testing on paired tumor and blood samples is effective for discriminating mutations derived from CH from germline mutations and somatic mutations in tumor cells.

## Background

Precision medicine is an emerging approach for providing patient-specific treatment based on genomic data using next generation sequencing (NGS). Reliable and accurate NGS technologies are essential in precision medicine (1). Several commercial genomic tests are available (Table 1). Each of these tests has different characteristics including the sequencing system; the number of tested genes; sample type such as tumor, blood, or paired specimens; and the curation and annotation system.

**Table 1:** Comparison of the genomic tests NCC-Oncopanel®, MSK-IMPACT®, Guardant360®, and FoundationOne CDx®.

**Platform**	**Target**	**Sample type**	**Genes tested**	**Turnaround time**	**Approval**
NCC-Oncopanel® (2)	Solid tumor	Tumor (FFPE) and blood (control)	114 genes	–	Japan-approved
MSK-IMPACT® (3)	Solid tumor	Tumor (FFPE) and blood (control)	468 genes	21 days	FDA-approved
Guardant360® (4)	Solid tumor	Blood (cfDNA)	73 genes (Point mutations)	7 days	–
FoundationOne CDx® (5)	Solid tumor	Tumor (FFPE)	324 genes	Within 14 days	FDA and Japan-approved

*FFPE, formalin fixation and paraffin embedding; cfDNA, cell-free DNA*.

Clonal hematopoiesis (CH) produces a detectable somatic clonal mutation that is commonly associated with clonal expansion of hematopoietic cells with age (6). As the use of NGS analysis has increased, studies have reported some discordances between genotyping results from tumor tissue and plasma specimens, which may be caused by tumor heterogeneity, variable shedding of tumor DNA into the plasma, and/or CH (7, 8). Here, we report a case of *TP53*-mutated CH in a patient with lung adenocarcinoma.

## Case Presentation

**Figure 1:**
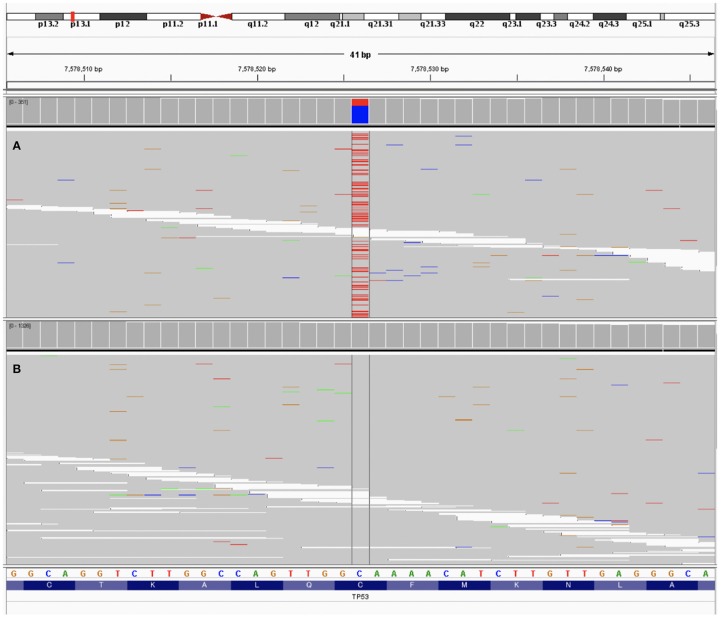
The data of patient's sample using next generation sequencing visualized by Integrative Genome Viewer (9). **(A)** Testing of the peripheral blood sample detected a *TP53* mutation (C135Y c404G>A [p.Cys135Tyr], allele frequency 29.8%). **(B)** Testing of the tumor tissue showed no *TP53* mutation.

A 74-year-old Korean man was referred to us for lung adenocarcinoma with left scapula metastasis. He had a normal complete blood count and no family history of malignant disease. Following palliative radiotherapy to the scapula, he had been treated with multi-line chemotherapies. However, his scapula metastasis had progressed. He provided written informed consent to undergo genomic testing. Undecalcified tumor tissue of the scapula metastasis from lung adenocarcinoma and a blood sample were submitted for NGS genomic testing using NCC-Oncopanel test, which was developed at the National Cancer Center in Japan. Testing of the blood sample detected a *TP53* mutation (C135Y c404G>A [p.Cys135Tyr], allele frequency 29.8%), but there was no matching mutation in the tumor tissue (Figure 1). The criteria for myelodysplastic syndromes (MDS), Waldenström macroglobulinemia, IgM monoclonal gammopathy of undetermined significance (IgM-MGUS), and Li-Fraumeni syndrome were not met. We suspected CH as the cause of the discordance between findings in tumor tissue and blood. We therefore verified the presence of the *TP53* mutation with a *TP53* target sequencing test using the sequencing by synthesis method (Falco Holdings Co., Ltd.) in blood and normal oral mucosa. The *TP53* mutation C135Y was only detected in the blood (Table 2).

**Table 2:** Results of genomic tests in a patient with *TP53*-mutated CH.

**Testing**	**Sample**	**Fresh or archived**	**Mutation**	**Results**
1st testing	Tumor Blood	Archived Fresh	No mutation *TP-53* mutation	Discordance between tumor and blood specimens
2nd testing	Oral mucosa Blood	Fresh Fresh	No mutation *TP-53* mutation C135Y c404G>A (p.Cys135Tyr)	Discordance between oral mucosa and blood specimens

*CH, clonal hematopoiesis*.

## Discussion and Conclusions

CH is an aging-related phenomenon in which hematopoietic stem cells or other early blood cell progenitors contribute to the formation of a genetically distinct subpopulation of blood cells (6). CH is considered to be associated with prior exposure to chemotherapy or irradiation in cancer patients as well as increased age (10). Plasma cell-free DNA genotyping detected that some mutations such as *JAK2, TP53*, and *KRAS* can be derived from CH and not from cancer (7).

The cause of the genotyping discordance between tumor tissue and blood is important for determining the treatment strategy. Although the *TP53* mutation is not targetable, germline *TP53* mutations result in a hereditary condition known as Li-Fraumeni syndrome and the *KRAS* mutation is a predictive marker of response to cetuximab therapy in colorectal cancer (11, 12). CH detected from cell-free DNA can cause misdiagnosis of the mutation origin and complicate the treatment strategy (7, 8). Pairing of plasma and tumor genomic tests is important, although paired sample testing is expensive.

We report a case of *TP53*-mutated CH in a lung adenocarcinoma patient in which target sequencing was used to confirm mutations derived from CH.

## Ethics Statement

The studies involving human participants were reviewed and approved by Genomic profiling study (The TOP-GEAR study) was registered at UMIN Clinical Trials Registry (UMIN 000011141) and approved by Institutional Review Board at National Cancer Center Hospital on June 27th, 2013. The patients/participants provided their written informed consent to participate in this study.

## Consent for Publication

This patient provided written informed consent to participate in TOP-GEAR study. The authors have obtained consent from the patient for publication of this case report.

## Author Contributions

MI and YF drafted the manuscript. YF contributed to the management of the clinical case. MI, YF, TKub, HM, TKum, TS, KS, NY, and TKo reviewed the manuscript and participated in clinical data interpretation. All authors read and approved the final manuscript.

### Conflict of Interest

YF reports speaker's bureau from Sysmex. NY is a recipient of a research grant from Japan Agency for Medical Research and Development (AMED, 17lk1403003h0001, and 18lk1403003h0002). KS and TKo are recipients of a collaborative research grant from the Sysmex Corporation. The remaining authors declare that the research was conducted in the absence of any commercial or financial relationships that could be construed as a potential conflict of interest.
